# Chitosan-Reinforced Gelatin Microspheres-Modified Glass Ionomer Cement (GIC): A Novel Bone Alloplast Graft Material Synthesis and an In Vivo Analysis

**DOI:** 10.7759/cureus.50384

**Published:** 2023-12-12

**Authors:** Sundaram Surendran, Subhashree Rohinikumar, Rajalakshmanan Eswaramoorthy, Karthik M, Thiyaneswaran Nesappan, Abhinav RP

**Affiliations:** 1 Prosthodontics and Implantology, Saveetha Dental College and Hospitals, Saveetha Institute of Medical and Technical Sciences, Chennai, IND

**Keywords:** guided bone regeneration, bone formation, nanoparticles, osseointegration, dental implants

## Abstract

Aim and objective

The study aimed to assess and evaluate the efficacy of glass ionomer modified with chitosan-reinforced gelatin microspheres on bone formation.

Materials and methods

The study involved three groups: Group I comprised plain glass ionomer cement; Group II comprised glass ionomer cement/gelatin (70:30 wt%); in Group III, glass ionomer cement/gelatin/chitosan (70:30%) scaffold were made into discs; the gelatin microspheres were synthesized by oil emulsion method. The synthesized scaffold was subjected to the following in vitro testing, Instron Universal Testing Machine (UTM), U3000, (Instron Corporation, Norwood, Massachusetts, United States) to assess compressive strength, scanning electron microscope (SEM) examination, and biocompatibility testing using hemocompatibility assay. The material was then tested in vivo; male Wistar albino rats, a total of nine animals, were utilized for this purpose. Three animals were used in each group; a femoral defect model was the model of choice for the experiment and the animals were observed for a period of four weeks, following which the animals were sacrificed and sent for histopathological analysis.

Results

The compression testing was carried out using UTM; test group I was 33 MPa, test group II was 2.3 MPa, and test group III was 25.75 MPa. SEM (JSM-IT800 Schottky Field Emission NANO SEM (JEOL, Tokyo, Japan)) analysis was done to observe the porosity of the fabricated scaffold with the average measurement of 0.12 ± 0.2 μm in test group II and 0.29 ± 0.4 μm in test group III. Hemocompatibility reports noted 0.4-0.8% lysis for the synthesized scaffolds. Histopathology staining of the femur defects showed that group III favoured bone formation. One-way analysis of variance (ANOVA) and post hoc Bonferroni test was done on the data. The optical density values of the alizarin red stained slide showed statistical significance for group III.

Conclusion

In conclusion, the synthesized scaffolds are biocompatible, distribution of porosity and pore characteristics in the glass ionomer cement/gelatin/chitosan group is better than that of the glass ionomer cement/gelatin group. The glass ionomer cement/gelatin/chitosan group had better compressive strength and induced more bone formation compared to the other test group and the control. Thus, the novel glass ionomer modified with chitosan-reinforced gelatin microspheres has optimal properties to be used as a bone graft material.

## Introduction

Dental implants have become an indispensable part of the treatment portfolio a dentist can offer to replace lost teeth; edentulism complete and or partial has been associated with significant loss of function, thus affecting the general health of the patient [1]. After the discovery of osseointegration and long-term clinical studies have proved the validity of this treatment modality [2], the tooth is usually housed in the alveolus of the jaws, which has a functional association with that of the dentition which is noticed based on the degeneration of the ridge after tooth loss; this resorption of the completely edentulous or partially edentulous alveolar ridge frequently jeopardises dental implant insertion in a prosthetically optimal position. As a result, before or in conjunction with implant placement, augmentation of a deficient bone volume is frequently required to achieve predictable long-term functionality and an aesthetically pleasing treatment result [3].

This calls for the use of autologous or synthetic substitutes that aid in the maintenance and or regeneration of the bone at the site of interest, by a guided tissue regeneration process. These substitutes tend to have complex manufacturing processes; in most cases, even though highly processed, these grafts tend to initiate the host donor's immune response, compromising the healing outcomes. Given the favourable nature of autograft or allograft on bone regeneration, the cost of these substitutes tends to be a major limiting factor when implant therapy is sought [4].

Many efforts have been made to understand and work with the complex requirements mandated for the bone substitute to be ideal and provide the most favourable outcome; a variety of materials have been used and are presently in use. Clinically, most of them are allogenic or xenogenic in origin, with a few alloplasts, which offer comparable results. Clinically, alloplasts have been used as fillers to augment large osseous defects and voids because of their osteoconductive nature and tend to be replaced in a very slow manner, remaining unaltered years together; alloplasts are less labour-intensive when it comes to the fabrication process and have the least risk for transmission of diseases. Most importantly, they have lesser cost when compared with adjuvants from other sources [5].

With the progressive use of dental implants, complication management becomes a part and parcel of the therapy and in most situations, there happens to be a bone loss around the implant, which would have to be augmented after thorough degranulation and disinfection. In such situations, the use of a graft or a biomaterial that would stay over a longer period would be the intervention of choice [6].

At present, the available xeno-allogenic graft material portfolio comes at a significant cost to the patient, and when autogenous bone graft is considered, the donor site morbidity and the patients' aversion to multiple surgical sites would produce a clinical standstill in the decision-making process for ridge augmentation procedures, thus naturally opening an avenue for the use of other alternatives, namely, the alloplastic graft solutions. Thus, current research is carried out in this field to develop novel substitutes to aid repair or regeneration.

The composition of various alloplasts used in medicine and surgery tend to have calcium sulphate or phosphate, bioactive glass, ceramic-based or biopolymer-based in their formulation [7] The sole purpose of this research paper is to develop a novel alloplastic bone substitute composed of commonly used dental cement; the glass ionomer restorative material has been modified with chitosan-reinforced gelatin microspheres and to study its mechanical properties, biocompatibility, and the degree of graft integration with the host tissue in animal models, we hypothesise that glass ionomer reinforced with chitosan-reinforced gelatin microspheres induces new bone formation [8-13]. Hence this study aims to assess and evaluate the efficacy of glass ionomer modified with chitosan-reinforced gelatin microspheres on bone formation.

## Materials and methods

Synthesis of chitosan-reinforced gelatin microspheres modified glass ionomer cement

Materials used for the synthesis of chitosan-reinforced gelatin microspheres have been listed in Table 1.

**Table 1:** Materials used in the fabrication of chitosan-reinforced gelatin microspheres modified glass ionomer cement

S.No	Materials
1	Gelatin (Hi-LR HIMEDIA)
2	Chitosan (HIMEDIA)
3	Acetone (RANKEM)
4	TWEEN 80 (LR GRADE RANKEM)
5	Olive Oil (Borges India)
6	Glass Ionomer Cement (Fuji, GC Corporation, Tokyo)

Phase 1: Fabrication of Gelatin Microspheres

**Figure 1:**
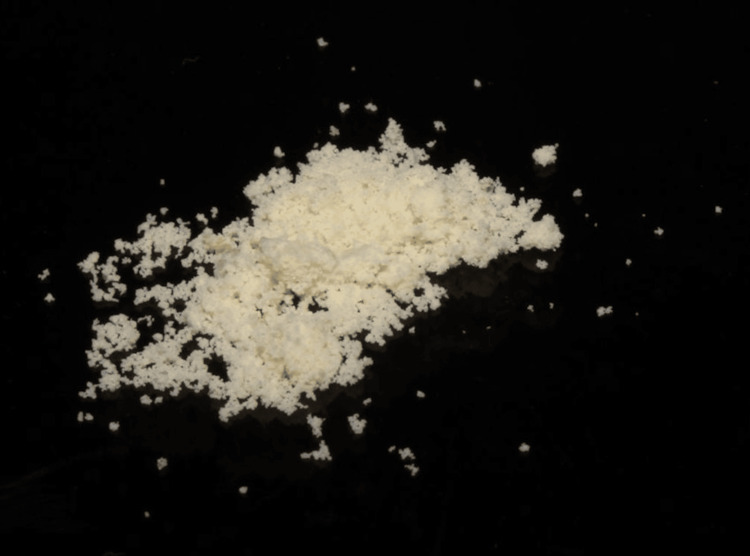
Synthesised gelatin microspheres

The gelatin microspheres were synthesized using water in an oil emulsion technique. Gelatin was dissolved in deionized water and added to an olive oil bath heated to 45° C while being continuously stirred. After 10 minutes the solution was cooled with additional stirring for 30 minutes, after which 40 ml of acetone was added and left for one hour. The microspheres were then cross-linked with 100 μL Tween 80 for 18 H while being stirred. The gelatin microsphere (Figure 1)** **thus fabricated was sieved and removed from the supernatant and washed with cooled acetone to remove remnant oil residue. The product was then freeze-dried overnight, weighed, and sterilised using a hot air oven.

Phase 2: Synthesis of Chitosan-Modified Gelatin Microparticle-Impregnated Glass Ionomer Scaffold

The fabricated gelatin microspheres and chitosan were weighed according to the concentrations required, 1% (individual weight proportion of 27 mg of chitosan, 270 mg of gelatin, and 2430 mg of glass ionomer cement); 3% (individual weight proportion of 71 mg of chitosan, 710 mg of gelatin, and 1890 mg of glass ionomer cement) and 5% (individual weight proportion of 135 mg of chitosan, 1350 mg of gelatin, and 1350 mg of glass ionomer cement), and taken on a glass slab; proportioned glass ionomer liquid was added and mixed till a homogeneous consistency was achieved. Then the paste form material was packed into a disc mould and allowed to set.

Phase 3: Fabrication of Porous Modified Glass Ionomer Scaffold Material

**Figure 2:**
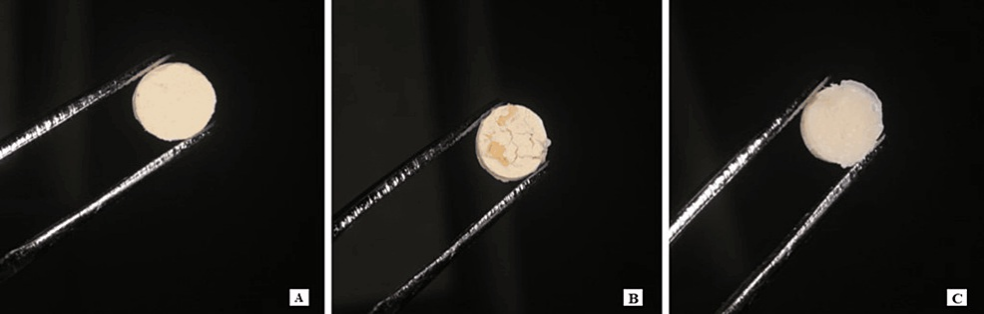
Scaffold discs; (A) glass ionomer, (B) glass ionomer cement 70% + gelatin 30%, (C) glass ionomer cement 70% + gelatin microspheres 30% + chitosan 5%

The retrieved disc was then placed in a water bath at 37° C for 60 minutes and removed and air dried; the discs were then sterilised in a hot air oven at 160° C for 60 minutes, packed in sterile autoclave pouches, vacuum sealed, and labelled according to the constituents (Figure 2).

In-vitro assessment of synthesized scaffolds

Compressive Testing

**Figure 3:**
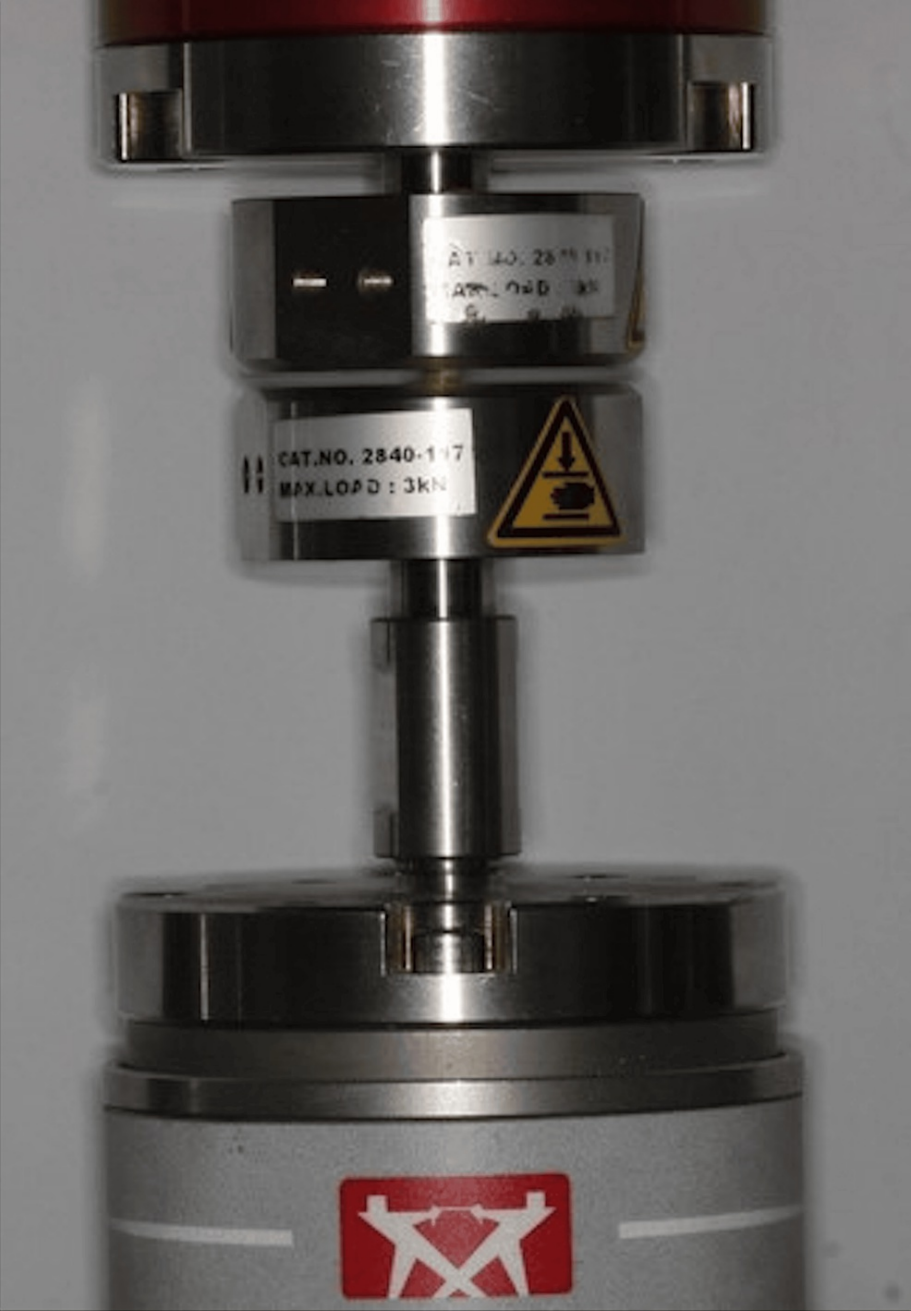
Synthesized scaffolds test in the UTM UTM: Universal Testing Machine (Instron Corporation, Norwood, Massachusetts, United States)

For testing the compressive strength of the sample used, an Instron Universal Testing Machine (UTM), U3000, (Instron Corporation, Norwood, Massachusetts, United States) (Figure 3) was used. The equipment rested on a flat table. It consisted of a lower member, an upper member, and a display board to display the amount of force applied. The apparatus was connected to a computer, which was used to record the pressure values and process data to provide the readings. During the test, various properties of the material were calculated and plotted as a stress-strain diagram in this situation to calculate the compressive stress at maximum force (MPa), compressive displacement at break (mm), compressive strain at break (%), and compressive stress at break (MPa). The test was carried out using platens or specialized fixtures on the machine. In principle, the test specimen was centrally positioned between the two parallel anvils and then compressed along its major axis at a constant rate of displacement until the specimen had deformed. Here the sample discs created were of size 6 x 3 mm. The discs were mounted into the specified plate. The upper crosshead arm of the universal testing machine once activated travels at a speed of 1 mm/minute parallel towards the lower arm of the machine, crushing it to measure the compressive strength of the sample. Force was recorded in Newton and was converted into MPa using the formula: CS= 4p/μd^2^, where, p = maximum force (N) and d = mean diameter of the specimen (mm).

Biocompatibility Assessment

**Figure 4:**
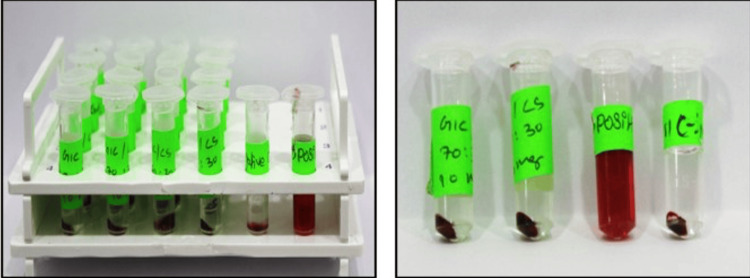
Vials containing blood depicting interactions with the synthesized novel biomaterial

A hemocompatibility test was conducted to rule out any potential adverse effects that the scaffold could produce. This way of testing the material has been presented as one of the most important criteria for the successful use of tested material in vivo. It has been incorporated in the ISO 10993-4 protocol for testing biomaterials and instrumentation that come and stay in close contact with blood. The process began with the collection of blood from a healthy volunteer in vials containing anticoagulant agent ethylene diamine tetra acetic acid (EDTA). The red blood cells were centrifuged at 4° C for 10 minutes and washed thrice with phosphate buffer saline (PBS, pH 7.4) to remove the plasma and other residues. A hemocompatibility assay was performed to estimate the red cell lysis rate in the presence of the scaffold and it was compared with negative control. The procedure was analysed with triplicates of all samples. All samples were incubated at 37° C for one hour. This sample was then centrifuged and the lysis rate was observed at a wavelength of 540 nm (Figure 4). The percentage was calculated using the formula hemolysis (%) = sample adsorbance - negative control x 100 positive control - negative control. And the results were tabulated.

Scanning Electron Microscope (SEM) Examination

SEM works at a high magnification. It reaches up to 300,000x and even 1,000,000x in advanced microscopes producing precise images of a wide variety of materials. Energy dispersive X-ray spectroscopy works together with SEM to provide qualitative and semiquantitative results. Thus, this synergistic test acts as an improved testing methodology for data far superior to dated routine laboratory investigations. At the forefront of precision imaging is the field emission SEM technology. This equipment uses lower accelerating voltages and small working distances to produce ultra-high-resolution images. SEM (JSM-IT800 Schottky Field Emission NANO SEM (JEOL, Tokyo, Japan)) was used. The sample discs were cleaned, removed of debris, and mounted on stubs using carbon tape for stabilization. Sputter coating was done. The analysis of the sections was done using a scanning electron microscope at ten- and fifty-micron magnification levels, using an electron beam which was produced by a current with a potential difference of 1kV.

In-vivo assessment of synthesized scaffolds

Study Setting

The present study was conducted in Saveetha Dental College and Hospitals, Chennai, India, after obtaining approval from the Institutional Animal Ethics Committee, and the study protocol met the requirements of the national guidelines of the Committee for Control and Supervision of Experiments on Animals. The study was given clearance with approval number BRULAC/SDCH/SIMATS/IAEC/05-2022/115. Wistar albino male rats, three to five months of age, 150-200 grams in weight, were used in the experiment.

Sample Size of Experimental Animals

The sample size was calculated from previously reported studies at an established power of 96% using G*Power (Heinrich Heine University Düsseldorf, Düsseldorf, Germany) statistics and estimated to be nine animals, three animals in each group.

Experimental Groups

The animals which satisfied the inclusion criteria were selected and allotted to the following study groups, as represented in Table 2.

**Table 2:** Table defining the different experimental groups

Group	Materials
Control Group	Conventional Glass Ionomer Cement (Group A)
Test Group 1	Conventional Glass Ionomer Cement + Gelatin Microspheres (Group B)
Test Group 2	Conventional Glass Ionomer Cement + Gelatin Microspheres + Chitosan (Group C)

Pretreatment Evaluation and Care for Animals

Quarantine refers to the isolation of the newly received animals from those already in the facility until the health and possible microbial status of the newly obtained animals have been determined. The newly procured Wistar albino rats were quarantined for one week to minimize the risk of the introduction of pathogens from these animals to animals already housed in the animal house. The animals were housed in a well-ventilated animal house, which was maintained at a constant temperature and a relative humidity of 55-65%. The animals were kept in spacious polypropylene cages and paddy husk was utilized as bedding material. The animals were maintained on a standard pellet diet and purified water, and bedding was changed frequently. The animal storage units were tagged with identification tags representing the different experimental groups.

Surgical procedures

Pre-surgical Preparation and Anaesthesia

The animals were washed using soap and water; the lateral side of the right thigh was shaved thoroughly and aseptically prepared with a solution of povidone-iodine. Rats are anaesthetized with ketamine hydrochloride (intraperitoneal (IP)) at the dosage of 75 mg/kg body weight and xylazine (intramuscular (IM)) at the dosage of 10mg/kg body weight. Meloxicam (subcutaneously) 1 mg/kg body weight was administered as pre-emptive analgesia.

Surgical Procedures: Femoral Defect and Graft Fixation

**Figure 5:**
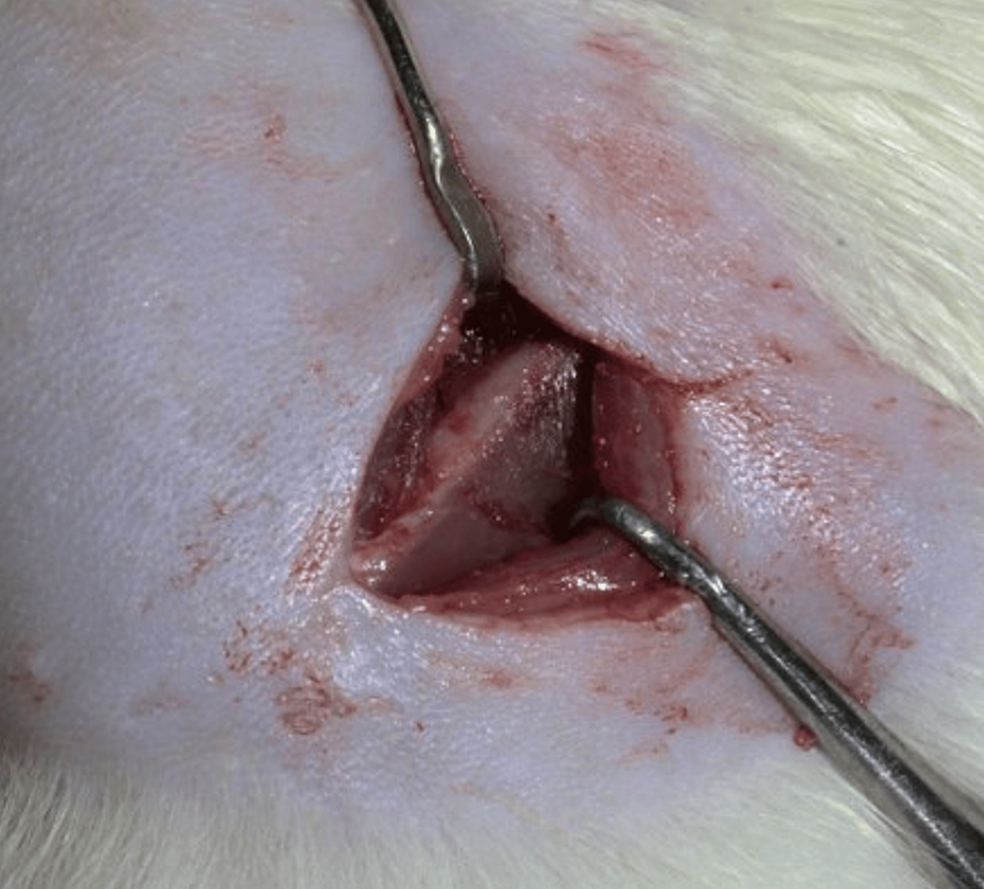
Identification of rat femur bone

**Figure 6:**
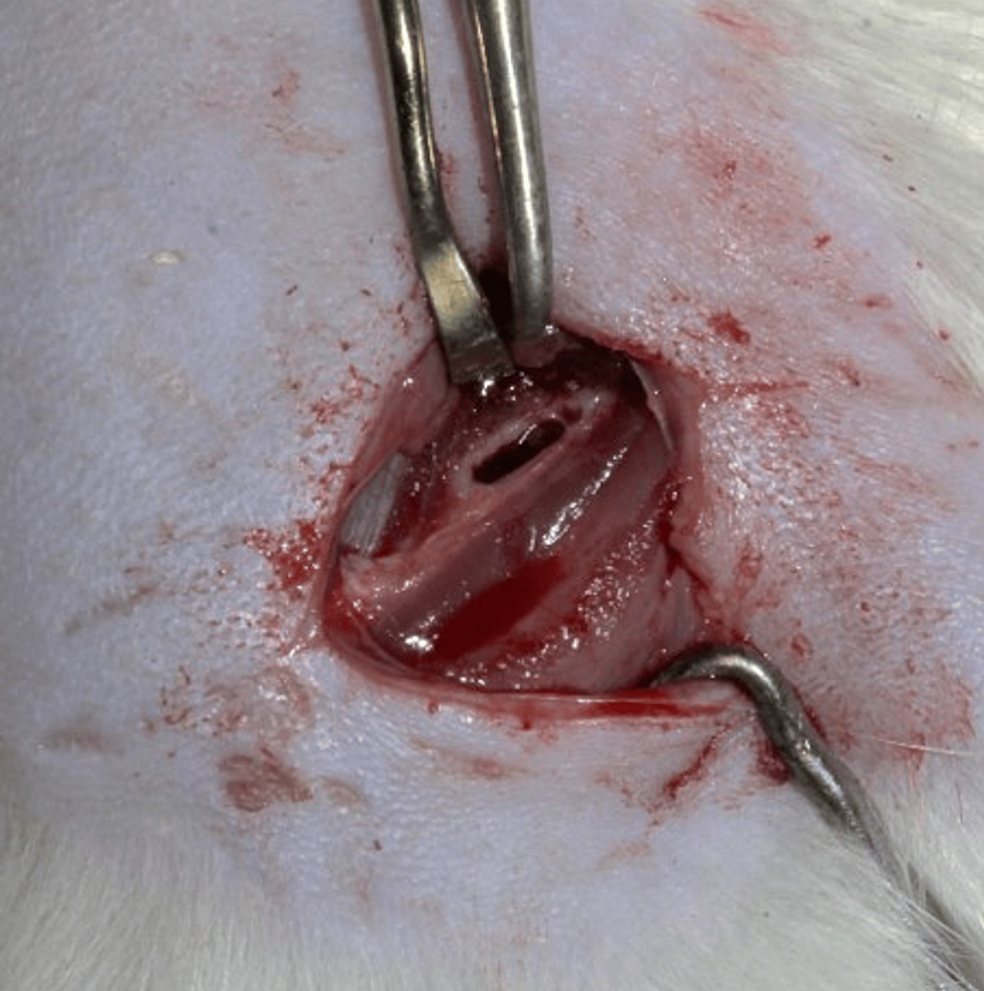
Critical size defect created in the rat femur bone

**Figure 7:**
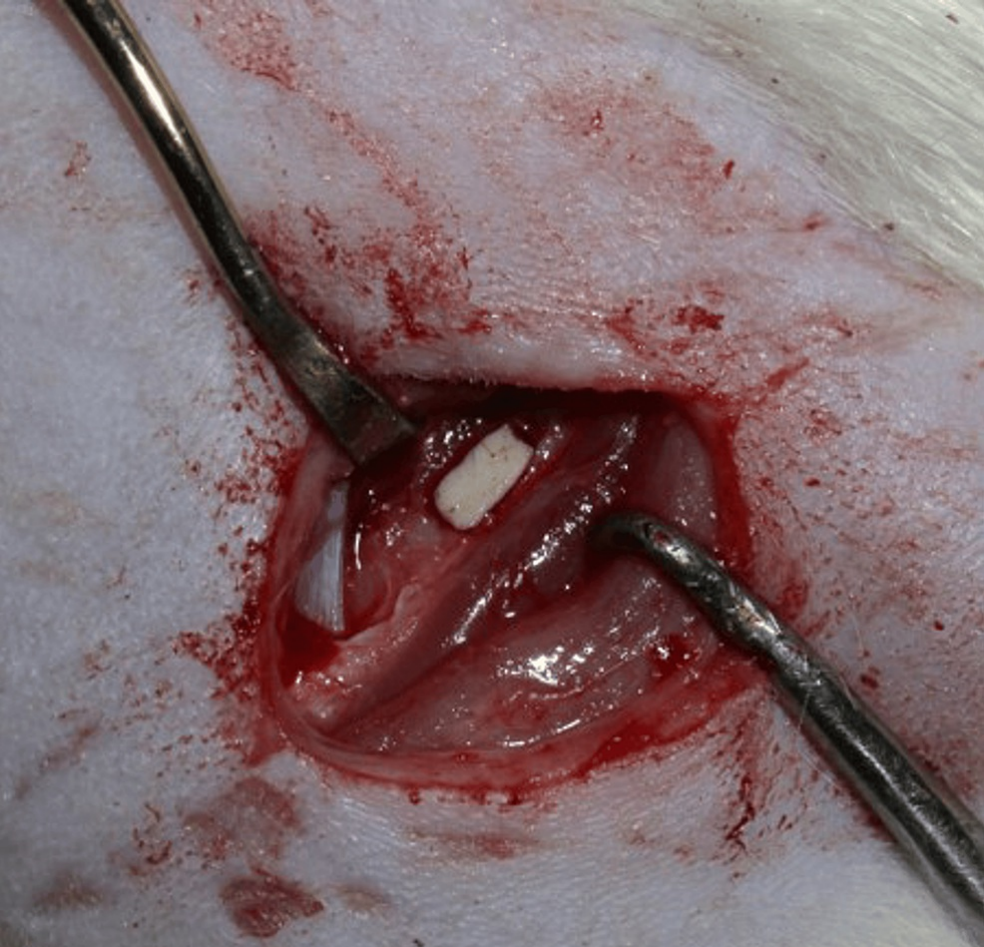
Placement and securing of the test graft material in the femoral critical size defect

A horizontal incision was made in the skin of the shaft of the femur about 1.5 cm in length. The skin and subcutaneous fascia were retracted and clamped. The septum and connective tissues in the space between the biceps femoris and the vastus lateralis muscles were cleaved and retracted, making the femoral shaft approachable (Figure 5). The osteotomy was initiated at the femur shaft region (1.5 mm depth and 8 mm length) using a trephine bur under copious continuous saline irrigation. The wound started to bleed once the marrow was reached, indicating the creation of the defect of the required depth. The bleeding was managed using cotton balls and the defect was extended linearly with a round trephine bur (Figure 6). The scaffold was adjusted according to the defect and was pushed into the defect. The graft was held in place through mechanical interlocking (Figure 7). Once satisfactory anchorage was achieved, the muscle and fascia were repositioned. It was done carefully to avoid damage to the blood vessels. The flaps were approximated using resorbable suture (Vicryl 5/0 (Ethicon®, Somerville, New Jersey, United States) and povidone-iodine was applied topically over the closed wound.

Post-surgical care and sample collection

The rats were housed in separate clean cages. The bedding was not placed after surgery to avoid infection. Meloxicam was continued for a complete course as a painkiller. The rats were examined daily for any inflammation or infection at the surgical site. The rats were continuously monitored for the entire study period of four weeks. At the completion of the study period, the animals were euthanized in a carbon dioxide chamber. Post mortem, the animals were transferred to the dissection tray, followed by incision and careful dissection to remove the entire femur. Then they were fixed in neutral buffered formalin and tagged according to the group. Then the specimens were sent for histopathological analysis.

## Results

Mechanical strength assessment

**Figure 8:**
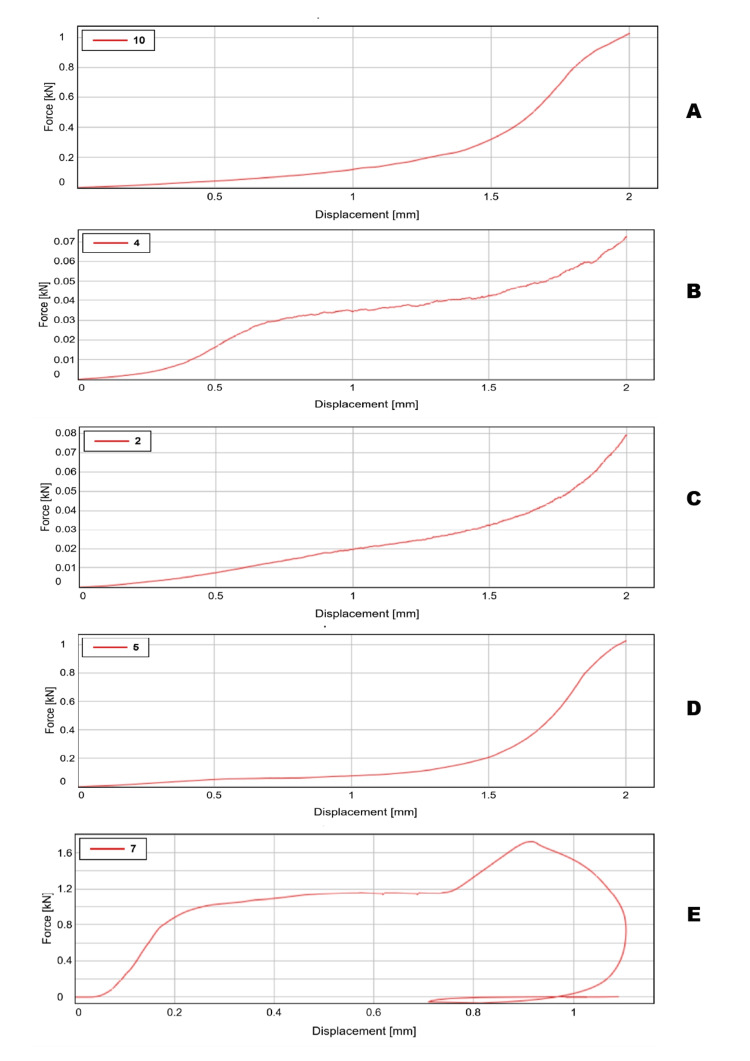
Graphical representation of compressive strength of glass ionomer cement and gelatin with various compositions In the graph, the X-axis represents the displacement of material (mm) and the Y-axis represents the force applied (KN). (A) Glass ionomer cement and gelatin 50:50 wt%; the maximum compressive strength was obtained at 21.51 MPa. (B) Glass ionomer cement and gelatin 70:30 wt%; the maximum compressive strength was obtained at 2.3 MPa. (C) Glass ionomer cement, gelatin, and chitosan 50:50 wt%; the maximum compressive strength was obtained at 3.6 MPa. (D) Glass ionomer cement, gelatin, and chitosan 70:30 wt%; the maximum compressive strength was obtained at 25.75 MPa. (E) Plain glass ionomer cement; the maximum compressive strength was obtained at 33 MPa.

The compression test values obtained from the Instron apparatus have been presented in Table 3, representing the various values obtained during the testing. Figure 8 represents the displacement curves of the biomaterials at various concentrations and the control group at maximum compression. On comparing all the groups, glass ionomer cement had the highest compressive strength, while doing the intergroup comparison. Glass ionomer cement 70% with 30% gelatin microspheres reinforced with 5% chitosan had higher compressive strength than the glass ionomer cement 70% with 30% gelatin microspheres group.

**Table 3:** The compressive strength (MPa) of the prepared biomaterial at various proportions (wt%) ^1^GIC: glass ionomer cement; ^2^GIC/GEL: glass ionomer cement 70% with 30% gelatin microspheres; ^3^GIC/GEL/CHS: glass ionomer cement 70% with 30% gelatin microspheres reinforced with 5% chitosan

Material	GIC^1^	GIC/GEL^2^		GIC/GEL/CHS^3^	
		50:50 wt%	70:30 wt%	50:50 wt%	70:30 wt%
Compressive Strength (MPa)	33	21.51	2.3	3.6	25.75

Biocompatibility

**Figure 9:**
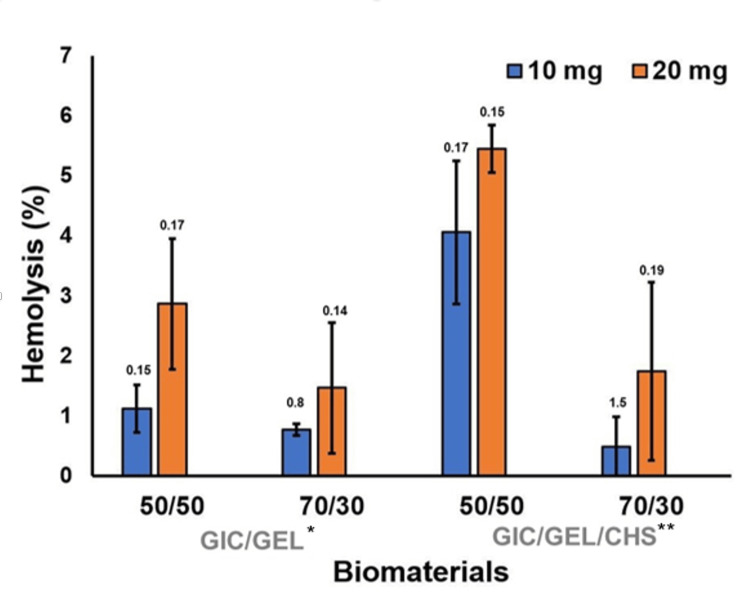
Hemocompatibility results obtained with different concentrations of graft material ^*^GIC/GEL: glass ionomer cement 70% with 30% gelatin microspheres; ^**^GIC/GEL/CHS: glass ionomer cement 70% with 30% gelatin microspheres reinforced with 5% chitosan

In the biocompatibility assay, the synthesized biomaterials had good agreement with blood cells. The data has been tabulated in Table 4 and graphically illustrated in Figure 9.

**Table 4:** Hemocompatibility results (lysis %) of synthesised biomaterial at various concentrations (wt%) Overall, the 50:50 wt% of both the test groups had higher lysis compared to 70:30 wt%. Ten mg of the test groups caused less lysis compared to 20 mg. The 70:30 wt% of glass ionomer cement/gelatin/chitosan had less lysis % at 10 mg concentration than the other groups. ^1^GIC/GEL: glass ionomer cement 70% with 30% gelatin microspheres; ^2^GIC/GEL/CHS: glass ionomer cement 70% with 30% gelatin microspheres reinforced with 5% chitosan

Biomaterial	GIC/GEL^1^		GIC/GEL/CHS ^2^	
	50:50 wt %	70:30 wt %	50:50 wt %	70:30 wt %
Lysis % at 10 mg	1	0.8	4	0.4
Lysis % at 20 mg	2.8	1.4	5.4	1.8

Surface characterization

**Figure 10:**
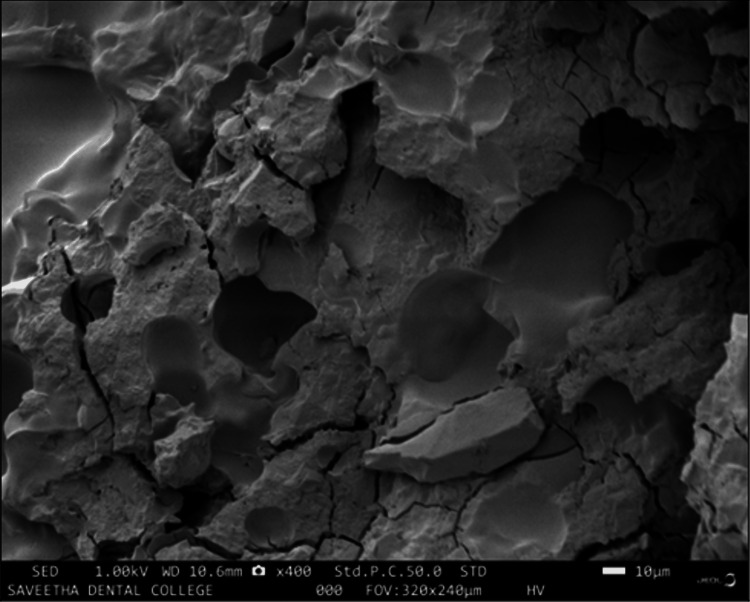
SEM image of plain glass ionomer cement The image of the sputter-coated plain glass ionomer cement sample magnified to 10 μm at 1.00 kV revealed a uniform dense glass ionomer matrix with an irregular surface, narrow cracks and surface crazing, along with agglomerates of unreacted particles.

**Figure 11:**
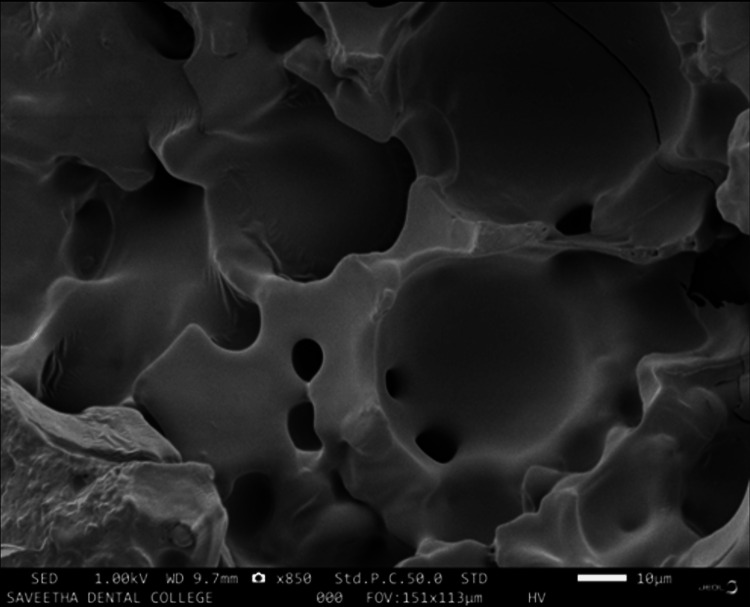
SEM image of glass ionomer cement 70% + gelatin microspheres 30% The image of the sputter-coated glass ionomer cement/gelatin microspheres modified (70:30 wt%) sample magnified to 10 μm at 1.00 kV revealed a matrix of glass ionomer cement with punched-out spherical spaces produced by the gelatin microspheres creating a network of lacunae like craters with uniformly distributed micro-porosities, with the size of the pore measured to be 0.12 ± 0.2 μm. SEM: scanning electron microscope

**Figure 12:**
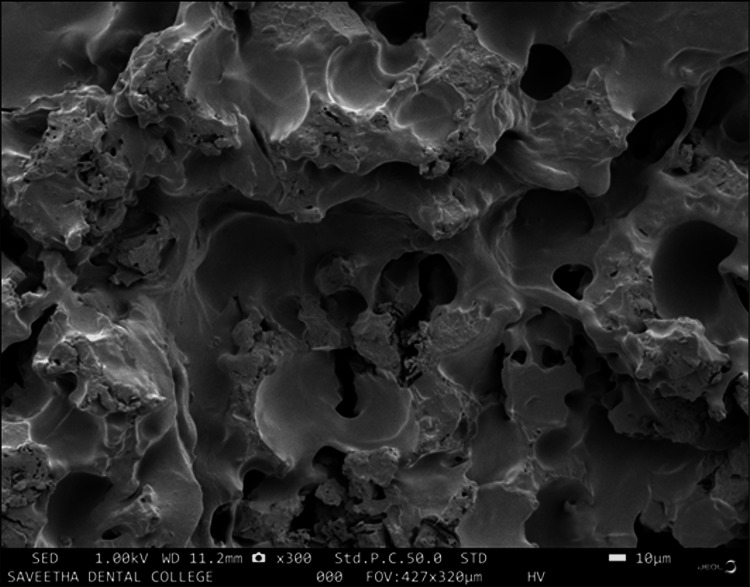
SEM image of glass ionomer 70% + gelatin microspheres 30% with 5% chitosan The image of the sputter-coated glass ionomer cement/gelatin microspheres modified (70:30 wt%) sample magnified to 10 μm at 1.00 kV revealed a glass ionomer matrix with a rough microtextured surface with fewer crater-like structures and regularly distributed micro-porosities surrounded by unreacted particles of chitosan; the micro-porosities measured to be 0.29 ± 0.4 μm. SEM: scanning electron microscope

When compared to the plain glass ionomer cement matrix (Figure 10), the SEM analysis points out the obvious micro-porosity formation following the inclusion of the nanoparticles (Figures 11, 12); the surface distribution of the porosities varied in between group B (0.12 ± 0.2 μm) and group C (0.29 ± 0.4 μm).

Histopathological assessment

**Figure 13:**
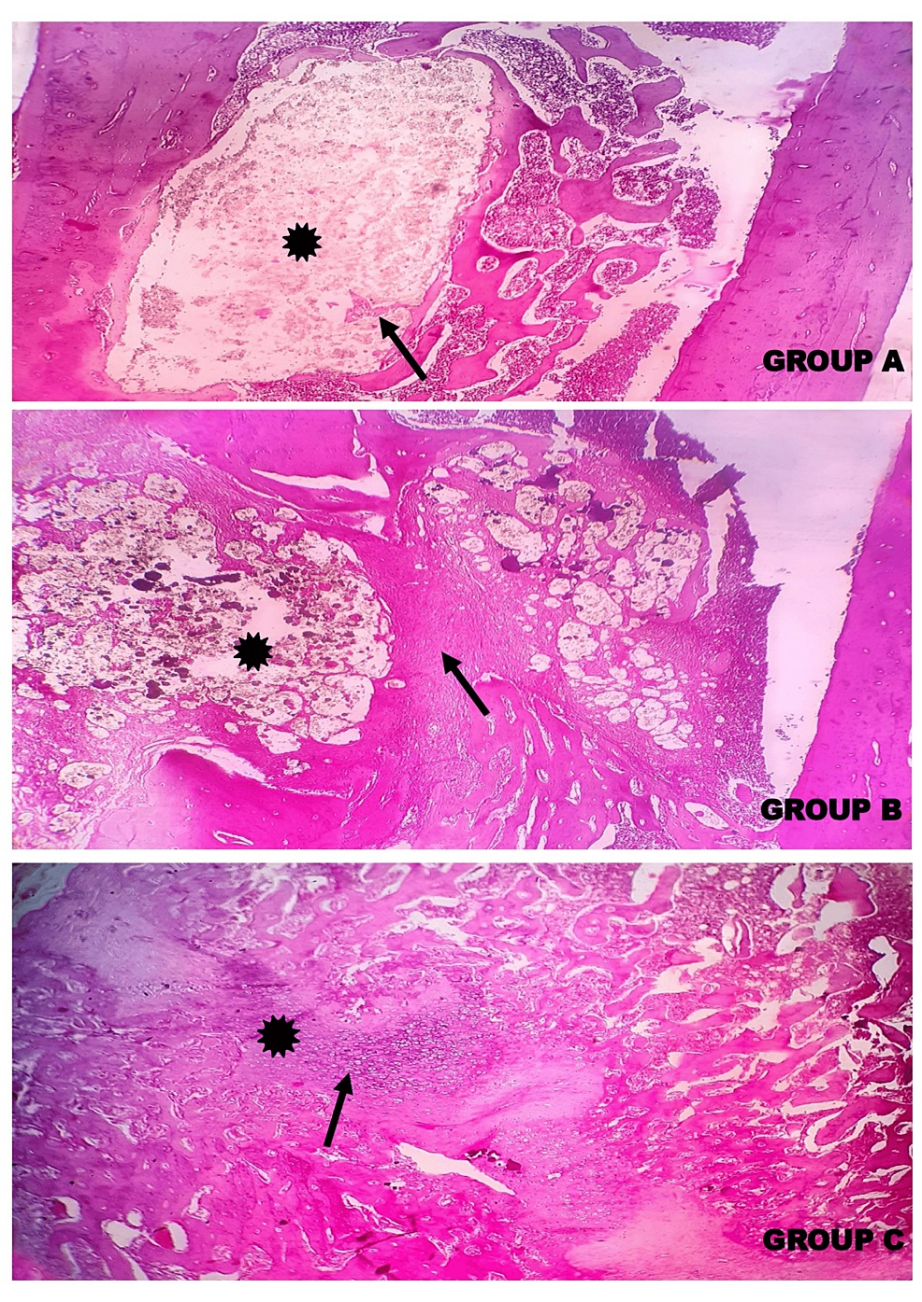
Photomicrographs showing the histopathology of Group A (Control) - GIC; Group B – GIC 70%, gelatin 30%; and Group C – GIC 70%, gelatin 30%, and chitosan 5% stained with H&E at 4X magnification The histopathological evaluation demonstrated the defect area (asterisk (*)) in between the two femoral shaft wall regions in control and other experimental groups. The formation of a network of woven bone (thin arrows (↑)) indicated the presence of immature bone at the defect area showing the course of bone remodelling, subsequent transformation, and maturation of the endochondral ossification in Group B and Group C. GIC: glass ionomer cement

**Figure 14:**
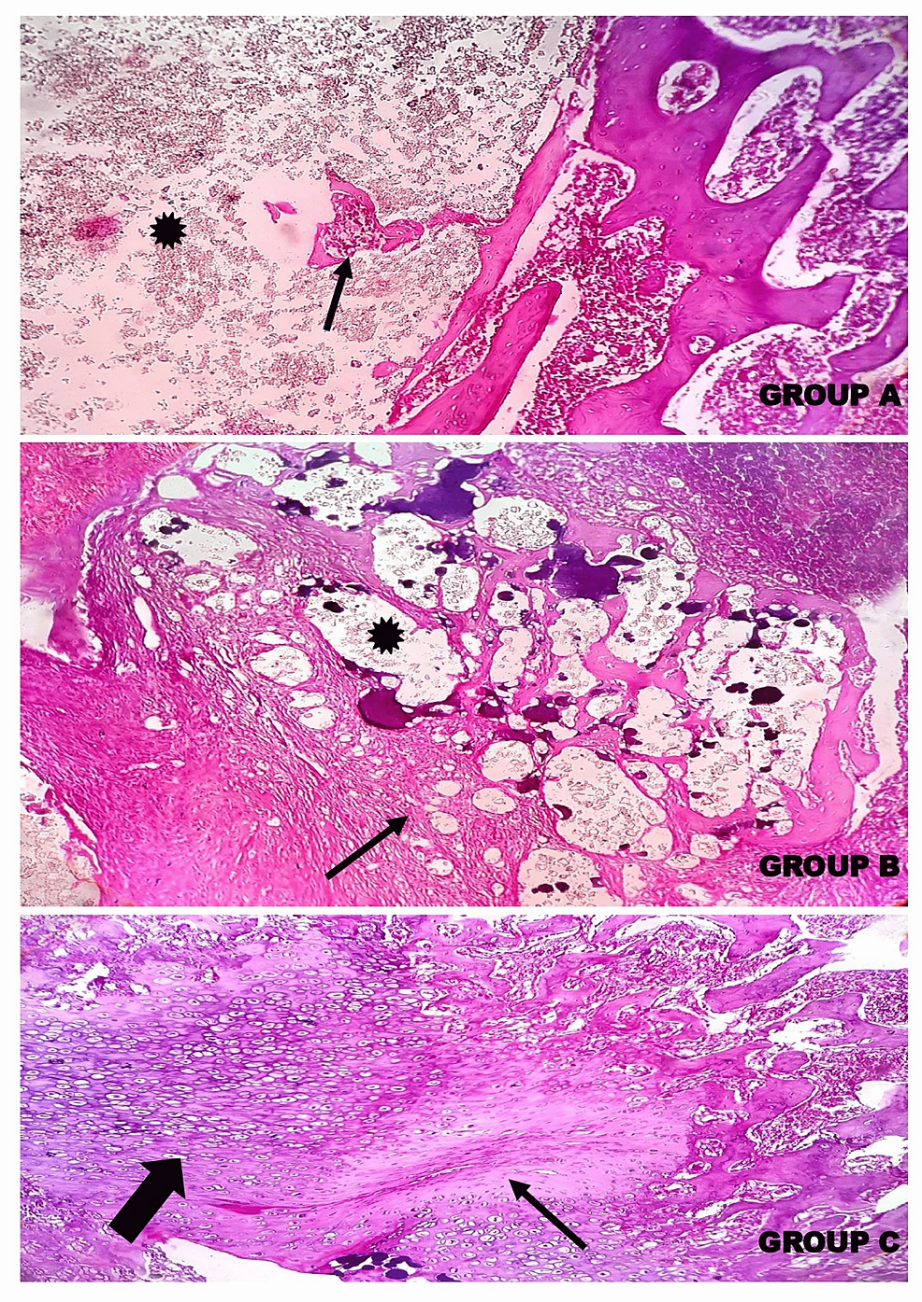
Photomicrographs showing the histopathology of Group A (Control) - GIC, Group B – GIC 70%, gelatin 30% and Group C – GIC 70%, gelatin 30%, chitosan 5% stained with H&E at 10X magnification. The histopathological evaluation demonstrated the defect area (asterisk (*)) in between the two femoral shaft wall regions in control and other experimental groups. The formation of a network of woven bone (thin arrows (↑)) indicates the presence of immature bone at the defect area, showing the course of bone remodelling and subsequent transformation and maturation of the endochondral ossification in Group A, Group B. In Group C, the callus shows the next phase of bone remodelling with the presence of chondrocytes transforming into osteocytes (thick arrow (↑)), which is not observed in Group A and Group B. GIC: glass ionomer cement

**Figure 15:**
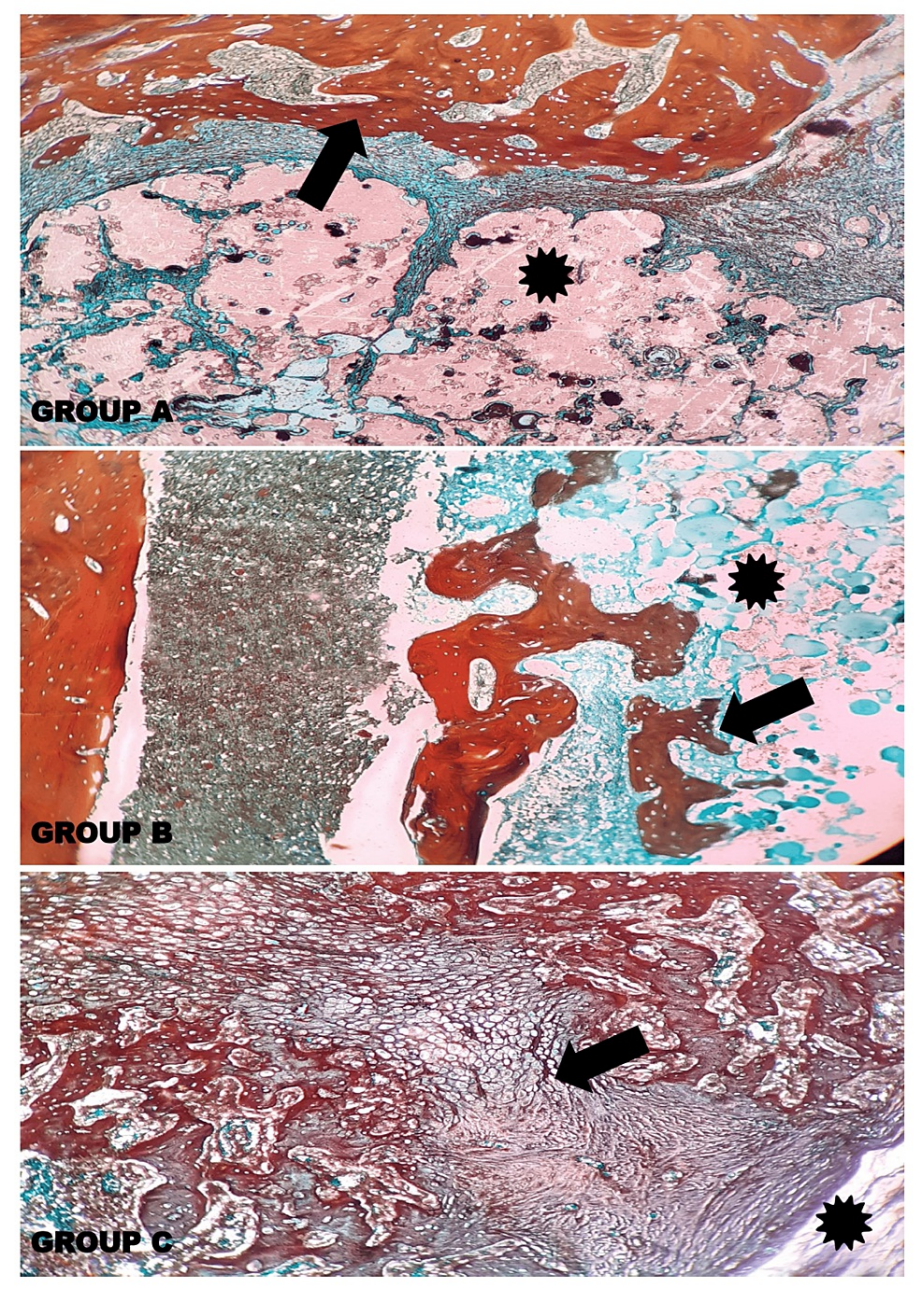
Photomicrographs showing the histopathology of Group A (Control) - GIC; Group B – GIC 70%, gelatin 30%; and Group C – GIC 70%, Gelatin 30%, Chitosan 5% stained with alizarin red stain at 10X magnification. The histopathological evaluation demonstrated the defect area (asterisk (*)) in control and other experimental groups. The formation of connective tissue fibres and a network of woven bone (thick arrows (↑)) in Group A, Group B, and Group C is depicted with a dark red colour. Non-osteogenic regions are dark green in colour. In Group C, most of the region imparts a dark red colour, depicting the presence of calcium deposition upon cellular transformation leading to calcified bone cells (thick arrow (↑)).

Histopathological evaluation at 5X and 10X magnification demonstrated the defect area in between the two femoral shaft wall regions in the control and other experimental groups. Woven bone formation was observed indicating the presence of immature bone at the defect area, showing the course of bone remodelling and subsequent transformation and maturation of the endochondral ossification in Group A, Group B, and Group C. In Group C, the callus showed the next phase of bone remodelling with the presence of chondrocytes transforming into osteocytes, which was not observed in Group A and Group B, which is more apparent in the alizarin red staining (Figures 13, 14, 15).

Optical density assessment

**Figure 16:**
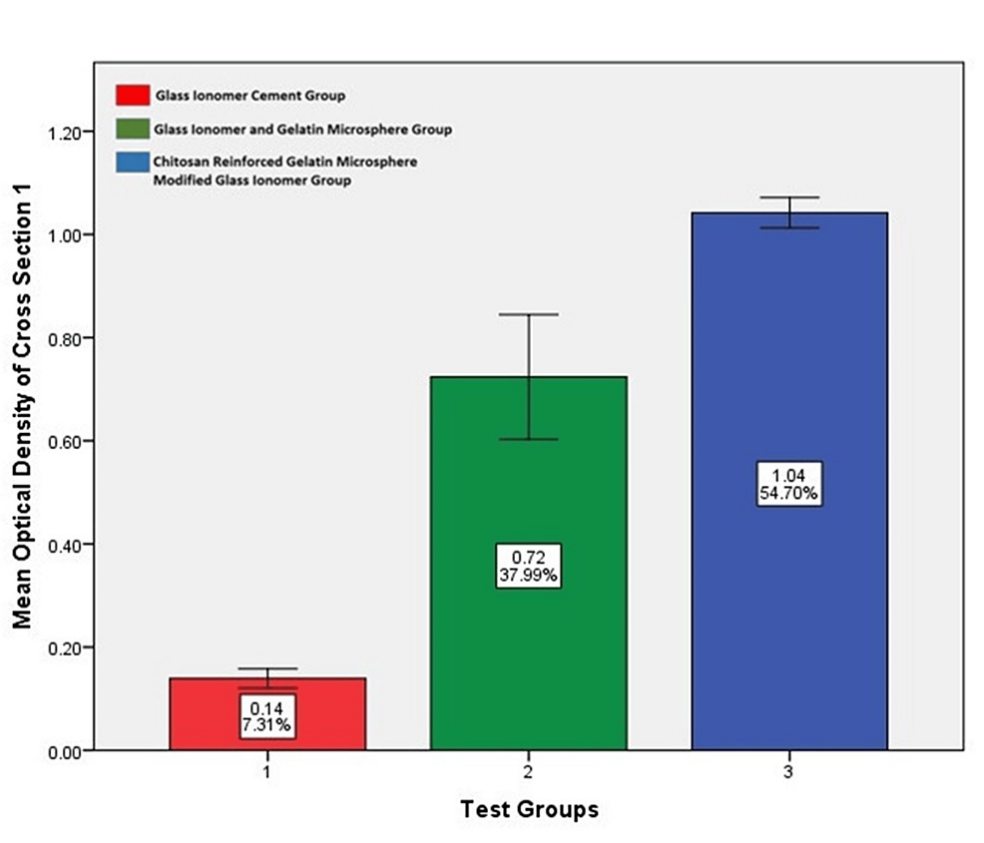
Mean optical density value among various groups in alizarin cross-section one as evaluated by ImageJ software* Chitosan-reinforced gelatin microsphere-modified glass ionomer group had more optical density value compared to the other test groups. *National Institutes of Health, Bethesda, Maryland, United States

**Figure 17:**
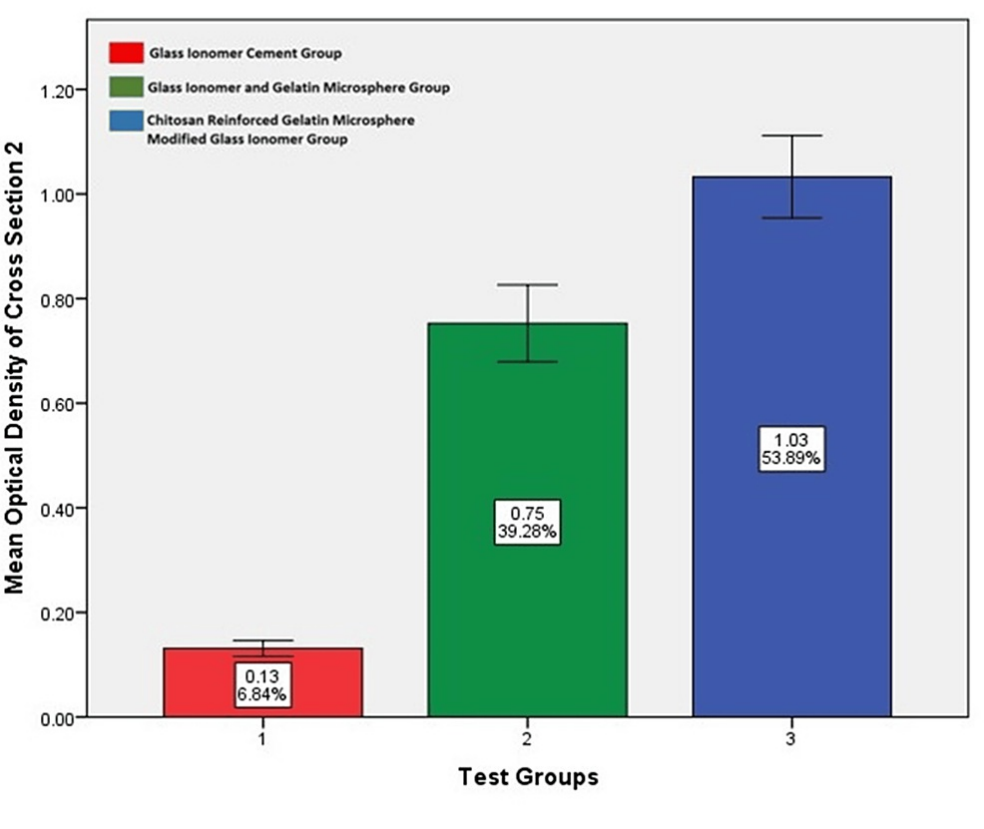
Mean optical density value among various groups in alizarin cross-section two as evaluated by ImageJ software* Chitosan-reinforced gelatin microsphere-modified glass ionomer group had more optical density value compared to the other test groups. *National Institutes of Health, Bethesda, Maryland, United States

**Figure 18:**
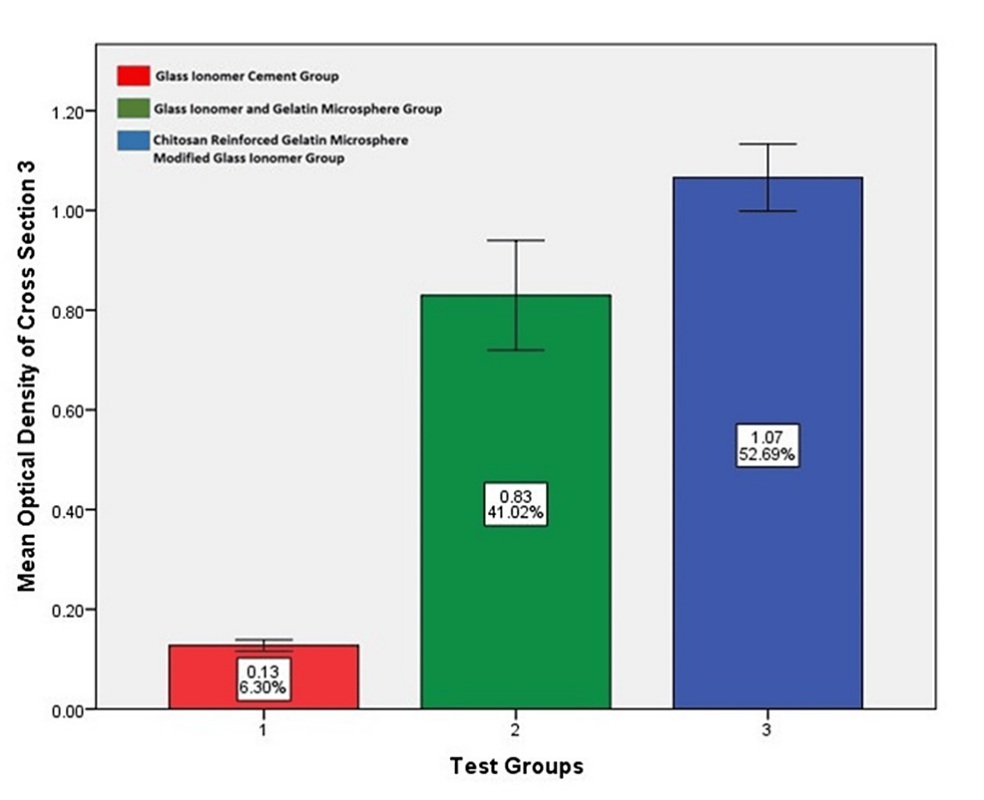
Mean optical density value among various groups in alizarin cross-section three as evaluated by ImageJ software* Chitosan-reinforced gelatin microsphere-modified glass ionomer group had more optical density value compared to the other test groups. *National Institutes of Health, Bethesda, Maryland, United States

The photomicrographs of alizarin red histopathology sections were evaluated using the ImageJ software (National Institutes of Health, Bethesda, Maryland, United States) to obtain the mean optical density of osseous tissue formed at the defect site. Using IBM SPSS Statistics for Windows, Version 29, (Released 2022; IBM Corp., Armonk, New York, United States), a one-way ANOVA test was done to compare differences, which yielded statistically significant results in test Groups II and III. On comparing the results between these two test groups using a Bonferroni post hoc analysis, test Group III had more bone formation. The results are given in Table 5 and illustrated graphically in Figures 16, 17, 18. Table 5 shows the optical density value of different groups. There is a statistically significant difference among the groups (p<0.05; one-way ANOVA) stating that the glass ionomer cement/gelatin/chitosan group had better bone formation than the other test groups (p<0.05; post hoc Bonferroni).

**Table 5:** Mean optical density values of the alizarin red stained samples of each test group on comparison using ImageJ Software** *One-way analysis of variance (ANOVA); 1: glass ionomer cement; 2: glass ionomer cement and gelatin microspheres; 3: chitosan-reinforced gelatin microsphere-modified glass ionomer group; 4: alizarin red stained sections of Group I, Group II, and Group III; **National Institutes of Health, Bethesda, Maryland, United States

Groups	Sample 1^4^		Sample 2^4^		Sample 3^4^		Significance*
	Mean	SD	Mean	SD	Mean	SD	
Group I – GIC^1^	0.139	0.32	0.13	0.26	0.12	0.019	0.00
Group II – GIC /GEL^2^	0.723	0.20	0.75	0.12	0.82	0.19	
Group III - GIC/GEL/ CHS^3^	1.04	0.05	1.03	0.13	1.06	0.11	

## Discussion

Bone grafts have become an integral part of implant dental practice, with a variety of procedures like ridge augmentation, sinus lift procedures, and in the management of bone defects and a variety of materials available in the market. Our aim was to develop a cost-effective material which would be biocompatible and have properties better than that of the available alloplastic material.

In this study, it was observed that the addition of gelatin microspheres significantly reduced the compressive strength, owing to the innate chondral nature, and the loss of the structural resilience due to the formation of microporosities. On the other hand, the addition of chitosan improved the mechanical strength; the synthesized graft material has a strength that falls on the scale of cancellous bone, similar to plain glass ionomer cement. The compressive strength is the most important property of the bone substitute material strength of the graft material. It is important to remember that the compressive strength of the human cortical bone ranges between 90 and 209 MPa and that of cancellous bone between 1.5 and 45 MPa [14]. The synthesized graft should match the compressive strength of the human bone. It is important that the graft materials coincide with this range so that the physiological process of bone remodelling and stress distribution occurs [15].

The scanning electron microscopy of the prepared scaffolds revealed that the gelatin microsphere-modified glass ionomer matrix had surface characters somewhat similar to that of cancellous osseous structures resembling interconnecting lacunae. This could be attributed to the evacuation of the microsphere after thermal treatment, leaving behind the spherical spaces and micropores. When this surface is compared to that of the chitosan-reinforced matrix where the lacunae are smaller and are surrounded by chitosan remnants around the microporosities, this microstructure attributes to the previously obtained compressive test values. Microporosities play a critical role in promoting the osteogenesis of scaffolds for bone tissue engineering; the inclusion of this physical feature in the graft material enhances cell attachment due to more protein binding sites [16]. The porosity acts as a zone for interaction between the scaffold and the bone cells. It also increases the surface area of the scaffold, thus generating enough capillary force to achieve penetration of host cells and blood [17]. The microporosity also aids the degeneration of the scaffold and the degeneration of products is further responsible for the progression of osteogenesis [18] and angiogenesis [19]. The inclusion of microporosities may tend to compromise the mechanical strength, but the quantum of the benefits is more when it comes to bone cell integration with the scaffold [20].

We evaluated the blood compatibility with 10 mg and 20 mg/mL of bioactive materials. The 70:30 wt% of the biomaterial at 10 mg/mL had a lower lysis rate compared to 50:50 wt% and 20 mg/mL of the biomaterial. Red blood cells' lysis percentage decreases in survival erythrocytes with the proportion of increasing concentration of the biomaterial. This material is observed to be biocompatible as well as biodegradable. Gelatin has been widely used in tissue engineering because of its biocompatibility, biodegradability and, non-immunogenicity [21] in addition to it being readily available, cost effective and the convenience of processing it to form substrate of interest [22]. Gelatin mimics the extracellular matrix of human tissues and organs [23]. Thus, microspheres of gelatin have been used as a vehicle to deliver cells and biologics in the field of regenerative medicine [24]. Chitosan has also been proven to be biocompatible; it has been reported that chitosan scaffold had excellent biocompatibility with no specific antibodies to the material as such. It was also observed that there was more collagen apposition and lesser immune cell infiltration, thus confirming it to be a non-immunogenic material. Chitosan is biodegradable and is frequently cross-linked in order to increase its functionality [25] as done in this experiment. Various experiments done on the biocompatibility yielded the same outcomes, proving chitosan scaffolds to be biocompatible.

It has the ability to promote the repair of tissues [26] and an innate antibiotic property owing to its cationic state, leading to ionic interaction with microbial cells. This, in turn, could be enhanced by adding additional agents also [27]. Conventional glass ionomer cement has been in use in dentistry for more than five decades. It is a versatile dental cement with a multifold application. Unlike its precursors, this dental material has a composition similar to that of bone excluding the silicate constituent. In vitro experiments revealed that conventional glass ionomer cement produced less alteration in human gingival fibroblast compared to resin-modified glass ionomer [28]. In a study with a similar outline, glass ionomer cement was modified with bioactive glass and subjected to osteoblastic cell culture assessment. It was observed that the modified cement was biocompatible and enhanced cell proliferation [29]. The animal tissue samples were subjected to histopathological analysis using two different stains, first with H&E staining, which clearly differentiates cartilage, bone and cellular elements, and then with alizarin red stain, which aids in assessing new bone formation by staining osteocytes and the osteoid matrix [30]. We could observe the formation of bone matrix and the influx of non-inflammatory cell infiltrates at the defect site. The processed slides were then processed using image processing software to highlight the zone of deposition. The optical density of the zones was calculated and tabulated. A sample containing a composite of glass ionomer, gelatin and chitosan 5% in the ratio of 70:30 wt% showed optimal deposition of the osseous matrix.

The limitation of the given study is the small sample size. Factors that could bias the study were the age and gender of the animal. This was overcome by standardising the animals; all three groups had rats three to five months of age and 150 to 200 grams in weight. The differences that could arise due to nutrition, diet, and environmental conditions among experimental animals were avoided by maintaining all the animals in a standard housing unit and feed. All the animals were maintained healthy and were closely monitored in the university animal house with utmost care for a period of one week before the procedure. The same operator with known experience handling small mammals had been employed to complete the procedure, thus reducing the risk of operator-associated discrepancies, and all the procedures were done in a strict aseptic protocol to avoid infection and inflammation that could have become a confounding factor. The presence of drugs or chemicals in an organism's environment could not have influenced bone formation, as the animals used in this study were medicated with equal doses of analgesics and antibiotics. The samples used in the surgery were also standardised before being placed into the osteotomy site, thus any errors that could happen on this aspect were avoided.

The findings of this study have paved the way for further scope of studies using the scaffold to assess bone formation characteristics in more complex defects and around implant surfaces. The scaffold could be further engineered to have more additional beneficial products such as growth factors and antimicrobial agents because of its nature. More studies can be conducted on this part using bigger sample sizes to generate more clinically relevant findings.

## Conclusions

Management of bone defects has become a common practice in implant dentistry, thus increasing the need for cost-effective biomaterials. Based on our observations, chitosan-reinforced gelatin microspheres-modified glass ionomer cement happens to be a viable alternative to existing material options and it happens to be biocompatible with adequate compressive strength, as well as micro-porosity traits characteristics, making it an ideal substrate for hard tissue regeneration. Experimentally, this material has improved bone formation in the animal bone defect model.
